# Targeting cell migration and the endoplasmic reticulum stress response with calmodulin antagonists: a clinically tested small molecule phenocopy of SEC62 gene silencing in human tumor cells

**DOI:** 10.1186/1471-2407-13-574

**Published:** 2013-12-05

**Authors:** Maximilian Linxweiler, Stefan Schorr, Nico Schäuble, Martin Jung, Johannes Linxweiler, Frank Langer, Hans-Joachim Schäfers, Adolfo Cavalié, Richard Zimmermann, Markus Greiner

**Affiliations:** 1 Department of Medical Biochemistry and Molecular Biology, Saarland University, Homburg, Saarland, Germany; 2 Department of Thoracic and Cardiovascular Surgery, Saarland University Hospital, Homburg, Saarland, Germany; 3 Experimental and Clinical Pharmacology and Toxicology, Saarland University, 66421 Homburg, Saarland, Germany

**Keywords:** Endoplasmic reticulum (ER) stress, Cell migration, Ca^2+^ homeostasis, Calmodulin antagonists, Sec62

## Abstract

**Background:**

Tumor cells benefit from their ability to avoid apoptosis and invade other tissues. The endoplasmic reticulum (ER) membrane protein Sec62 is a key player in these processes. Sec62 is essential for cell migration and protects tumor cells against thapsigargin-induced ER stress, which are both linked to cytosolic Ca^2+^. *SEC62* silencing leads to elevated cytosolic Ca^2+^ and increased ER Ca^2+^ leakage after thapsigargin treatment. Sec62 protein levels are significantly increased in different tumors, including prostate, lung and thyroid cancer.

**Methods:**

In lung cancer, the influence of Sec62 protein levels on patient survival was analyzed using the Kaplan-Meier method and log-rank test. To elucidate the underlying pathophysiological functions of Sec62, Ca^2+^ imaging techniques, real-time cell analysis and cell migration assays were performed. The effects of treatment with the calmodulin antagonists, trifluoperazine (TFP) and ophiobolin A, on cellular Ca^2+^ homeostasis, cell growth and cell migration were compared with the effects of siRNA-mediated Sec62 depletion or the expression of a mutated *SEC62* variant *in vitro*. Using Biacore analysis we examined the Ca^2+^-sensitive interaction of Sec62 with the Sec61 complex.

**Results:**

Sec62 overproduction significantly correlated with reduced patient survival. Therefore, Sec62 is not only a predictive marker for this type of tumor, but also an interesting therapeutic target. The present study suggests a regulatory function for Sec62 in the major Ca^2+^ leakage channel in the ER, Sec61, by a direct and Ca^2+^-sensitive interaction. A Ca^2+^-binding motif in Sec62 is essential for its molecular function. Treatment of cells with calmodulin antagonists mimicked Sec62 depletion by inhibiting cell migration and rendering the cells sensitive to thapsigargin treatment.

**Conclusions:**

Targeting tumors that overproduce Sec62 with calmodulin antagonists in combination with targeted thapsigargin analogues may offer novel personalized therapeutic options.

## Background

Cancer is one of the most common deadly diseases [1], and the proportion of patients dying because of malignant disease is increasing every year [2]. Lung cancer is of particular concern with a five-year survival rate below 20% [3]. Therapeutic opportunities are scarce for patients suffering from squamous cell carcinoma (SCC) of the lung [4]. We have recently reported *SEC62* as a new candidate oncogene, as it is significantly overexpressed with elevated protein levels in SCC [5].

Sec62 is an essential protein in yeast and part of the Sec62/Sec63 sub-complex of the SEC complex, acting as a docking site for posttranslational protein transport [6]. Studies in mammals have shown that Sec62 is associated with the heterotrimeric Sec61 complex and Sec63 [7,8], and that it participates in the targeting and translocation of small pre-secretory proteins to the endoplasmic reticulum (ER) [9,10]. Mammalian Sec62 can also interact with the ribosome, thereby regulating translation [11]. Elevated Sec62 protein levels are functionally linked to increased cell migration capability [12] and reduced sensitivity to thapsigargin-induced ER stress [13], both of which are tightly regulated by the cytosolic Ca^2+^ concentration [14-16]. Previously, we have shown that reduced Sec62 protein levels lead to an at least two-fold increase in basal cytosolic Ca^2+^ and a much greater increase in cytosolic Ca^2+^ concentration in response to thapsigargin treatment (*i.e.*, increased ER Ca^2+^ leakage) [13]. These results demonstrate a significant influence of Sec62 on ER Ca^2+^ homeostasis, making Sec62 a promising target for new therapeutic approaches. Regulation of cytosolic Ca^2+^ levels by targeting this protein may induce anti-metastatic and anti-proliferative effects.

In the present study, we used small molecule inhibitors of the Ca^2+^-binding protein, calmodulin, to mimic the phenotypes previously observed after *SEC62* silencing. This approach provided new insight into the physiological function of Sec62 and may lead to a new therapeutic strategy for personalized cancer therapy.

## Methods

### Cell culture and tissue samples

PC3 (DSMZ no. ACC 465), HeLa (DSMZ no. ACC 57), A549 (DSMZ no. ACC 107), BC01 (kindly provided by G. Unteregger, Saarland University Hospital, Department of Urology and Pediactric Urology), BHT 101 (DSMZ no. ACC 279), ML1 (DSMZ no. ACC 464) and HEK293 (DSMZ no. ACC 305) cells were cultured at 37°C in DMEM medium (Gibco Invitrogen, Karlsruhe, Germany) containing 10% fetal bovine serum (FBS; Biochrom, Berlin, Germany) and 1% penicillin/streptomycin (PAA, Pasching, Austria) in a humidified environment with 5% CO_2_. H1299 cells (ATCC no. CRL-5803D) were cultured in RPMI1640 medium (PAA) containing the same supplements. We used stably transfected HEK293 cells expressing plasmid-encoded wild-type *SEC62* (p*SEC62*-IRES-GPF) or an empty control plasmid (pIRES-GPF) [5]. A plasmid encoding *SEC62* with a D308A point mutation (p*SEC62*_*D308A*_-IRES-GPF) was generated using the QuikChange Site-Directed Mutagenesis Kit (Stratagene, La Jolla, CA, USA) according to the manufacturer’s instructions. The plasmid was sequenced to confirm the point mutation. A stably transfected cell line expressing this mutant gene was generated by transfecting 2.4 × 10^5^ HEK293 cells in a 6-well plate using FuGeneHD Reagent (QIAGEN, Hilden, Germany) according to the manufacturer’s instructions. After 72 h, the medium was replaced with normal culture medium containing 1% G418 and the cells were cultured until selection was achieved. After harvesting, the cells were diluted to a density of 1 cell per 100 μl, and 100 μl were seeded in each well of a 96-well plate in medium containing 1% G418. Clones originating from a single cell were selected and analyzed for Sec62 content. All experiments using stably transfected cell lines were performed in normal growth medium containing 1% G418. Stably transfected HEK293 cells were used for migration assays, as transient transfection or treatment with FuGeneHD transfection reagent strongly inhibits cell migration.

We analyzed Sec62 levels in cancerous and tumor-free lung tissue from 70 non-small cell lung cancer (NSCLC) patients with pathologically confirmed adenocarcinoma (AC) or squamous cell carcinoma (SCC) using western blot with β-actin as a loading control. We calculated the relative elevation in the Sec62 protein content (rSec62 = [Sec62_tumor_/b-actin_tumor_]/[Sec62_tumor-free_/b-actin_tumor-free_]) in the tumor [5]. All patients (n = 70) and the subgroups of AC (n = 35) and SCC (n = 35) patients were divided into two groups based on the median rSec62 value, and survival analyses were performed using the Kaplan-Meier method and the log-rank test. Only samples from patients who gave signed informed consent were used. All samples were received for therapeutic or diagnostic purpose and anonymized. Therefore, according to the guidelines of the local ethics board (“Ethikkommision der Ärztekammer des Saarlandes”) and the statement of the national ethics committee (nationaler Ethikrat (Hrsg.): Biobanken für die Forschung. Stellungnahme. Berlin 2004 [http://www.ethikrat.org/dateien/pdf/NER_Stellungnahme_Biobanken.pdf]) they can be used without specific approval by an ethics board.

### Western blot

Protein in lysates from 2 × 10^5^ cultured cells was quantified by western blot analysis. We used an affinity-purified polyclonal rabbit anti-peptide antibody directed against the C-terminus of human Sec62, a polyclonal rabbit anti-BiP antibody, a polyclonal rabbit anti-peptide antibody directed against the C-terminus of human Sec61α, and a monoclonal murine anti-β-actin antibody (Sigma Aldrich, Taufkirchen, Germany, A5441-.5ML). The primary antibodies were visualized using an ECL^TM^ Plex goat anti-rabbit IgG-Cy5 or ECL^TM^ Plex goat anti-mouse IgG-Cy3 conjugate (GE Healthcare, Munich, Germany), and the Typhoon-Trio imaging system (GE Healthcare) in combination with Image Quant TL software 7.0 (GE Healthcare). We determined the ratio of Sec62, Sec61α and BiP relative to β-actin.

### Silencing of gene expression by siRNA

For gene silencing, 5.4 × 10^5^ cells were seeded in 6-cm dishes containing normal culture medium. The cells were transfected with *SEC62*-UTR siRNA (CGUAAAGUGUAUUCUGUACtt; Ambion, Life Technologies, Carlsbad, CA, USA), *SEC62* siRNA (GGCUGUGGCCAAGUAUCUUtt; Ambion), *SEC61A1* siRNA (GGAAUUUGCCUGCUAAUCAtt, QIAGEN, Hilden, Germany), or control siRNA (AllStars Neg. Control siRNA; QIAGEN) using HiPerFect Reagent (QIAGEN) according to the manufacturer’s instructions. After 24 h, the medium was changed and the cells were transfected a second time. Silencing efficiency was evaluated by western blot analysis. The maximum silencing effect was seen 72 h (*SEC62* siRNAs) or 96 h (*SEC61A1* siRNA) after the first transfection.

### Real-time cell proliferation analysis

The xCELLigence SP system (Roche Diagnostics GmbH, Mannheim, Germany) was used for real-time analysis of cell proliferation. In this system, 1.0 × 10^4^ or 2.0 × 10^4^ stably transfected HEK293 cells, untreated HEK293, PC3 or HeLa cells, or PC3 cells pretreated with siRNA in 6-cm dishes were seeded into a 96-well e-plate (Roche Diagnostics GmbH) according to the manufacturer’s instructions. Cells pretreated with siRNA were seeded 24 h after the second transfection. When cells were treated with thapsigargin, TFP or ophiobolin A, the treatment was performed at least 4 h after seeding the plates. Cell proliferation was monitored for 53–96 h and the data was evaluated with RTCA 1.2 software (Roche Diagnostics GmbH). Thapsigargin was used at concentrations of 6 or 10 nM, because these concentrations did not affect cell growth. This is in contrast to the live-cell calcium imaging experiments, where 1 μM thapsigargin was used to visualize short-term calcium effects monitored only over a time span of up to 1200 s.

### Peptide spot binding assay

Thirteen peptides spanning the N-terminus of the human Sec61α protein were synthesized on cellulose membranes via a C-terminal attachment as described previously [17,18]. The peptides consisted of 12 amino acid residues with an overlap of 10 residues and were incubated in binding buffer (30 mM Tris–HCl, pH 7.4, 170 mM NaCl, 6.4 mM KCl, 5% sucrose, 0.05% Tween20) with Sec62-C-6His (1 μM), which was purified from *Escherichia coli* as described previously [11]. To detect bound protein, the membranes were washed twice with binding buffer, incubated with anti-His-POD-coupled antibody (1:1000, QIAGEN), washed twice with binding buffer again, incubated with ECL (GE Healthcare) and visualized using a lumi-imaging system (Roche Diagnostics GmbH).

### Surface plasmon resonance spectroscopy

Surface plasmon resonance (SPR) spectroscopy was performed in a BIAlite upgrade system (Biacore, Freiburg, Gerrmany). Peptides representing the N-terminus of Sec61 (AIKFLEVIKPFC) or the N-terminus of TRAM (VLSHEFELQNGADC) were immobilized in the measuring cell or control cell, respectively, on a CM5 sensor chip using ligand-thiol-coupling according to the manufacturer’s protocol. Measurements were performed at a flow rate of 10 μl/min in a Ca^2+^−free buffer containing 10 mM HEPES-KOH, pH 7.4, 150 mM NaCl, 2 mM MgCl, 6.4 mM KCl and 0.005% surfactant. For interaction analysis, *E. coli*-purified Sec62-C-6His (1 μM) [11] in buffer minus Ca^2+^ or in the same buffer containing 2 mM Ca^2+^, or the Ca^2+^-containing buffer alone was passed over the chip. Response units are shown as the difference between the measuring and control cells. The analysis was carried out using BIA evaluation software version 3.1 (Biacore) with 1:1 binding models and mass transfer.

### Migration potential analysis

Migration was tested using the BD Falcon FluoroBlok system (BD, Franklin Lakes, NJ, USA) in 24-well inserts. A total of 2.5 × 10^4^ stably transfected HEK293 cells, or untreated PC3 or HeLa cells were loaded in normal medium containing 0.5% FBS. When DMSO, TFP or ophiobolin A was used, the drugs were added to the top and bottom chambers at various concentrations. The inserts were placed in medium with 10% FBS as a chemoattractant. After 72 h, the cells were fixed with methanol and stained with DAPI, and migrating cells were analyzed on the back of the membrane using fluorescence microscopy.

### Live-cell calcium imaging

For live-cell Ca^2+^ imaging, HeLa cells were loaded with 4 μM FURA-2 AM (Molecular Probes, Eugene, OR, USA) in DMEM for 45 min at room temperature as described previously [19,20]. Two washes were performed with a Ca^2+^-free buffer (140 mM NaCl, 5 mM KCl, 1 mM MgCl_2_, 0.5 mM EGTA and 10 mM glucose in 10 mM HEPES-KOH, pH 7.35) and the experiments were carried out in the same solution. A ratiometric measurement was performed for 3 min to determine the initial cytosolic [Ca^2+^]. The measurement was continued after the addition of 1 μM thapsigargin or - to measure store operated calcium entry (SOCE) - 2.5 mM Ca^2+^. Cells pretreated as described in the text were compared with respect to the initial cytosolic [Ca^2+^] and thapsigargin-induced changes in cytosolic [Ca^2+^]. Data were collected by an iMIC microscope and polychromator V (Till Photonics, Graefelfing, Germany) by alternating excitation between 340 and 380 nm, and measuring the emitted fluorescence at 510 nm (dichroic, DCLP410; emitter filter LP470; Till Photonics). Images containing 50–60 cells/frame were sampled every 3 sec. FURA-2 signals were recorded as an F340/F380 ratio, where F340 and F380 correspond to the background-subtracted fluorescence intensities at 340 and 380 nm, respectively. The cytosolic [Ca^2+^] was estimated from the ratio measurements using an established calibration method [21].

ER luminal Ca^2+^ was determined using HeLa-CES2 cells that contain ER lumenal carboxylesterase and allow efficient dye loading of the ER, as previously described [22]. Cells were loaded with 4 μM Fluo5N AM (solubilized in Pluronic F-127) in HBSS (Gibco) for 15 min at 37°C, washed with HBSS and incubated for another 30 min at 25°C to remove remaining cytosolic dye. After 1 min incubation in Ca^2+^-free buffer, buffer (0.1% DMSO, solvent control), ophiobolin A (100 μM) or TFP (10 μM) were added, samples were measured for 2 min, and then 1 μM thapsigargin was added to unmask the passive Ca^2+^ efflux from the ER. After 8 min, 5 μM ionomycin was applied to release the total ER Ca^2+^ of the cells. Data were collected by the iMIC microscope with excitation at 490 nm and measurement of the emitted fluorescence at 530 nm. Images containing 10–25 cells/frame were sampled every 3 s. A τ_1/2_-value was calculated for each curve as the time point at which 50% reduction of fluorescence signal was achieved after addition of thapsigargin.

Data were analyzed using Excel 2007 and Origin 6.1.

## Results

### Sec62 levels in cancer tissue predicts survival of NSCLC patients

**Figure 1:**
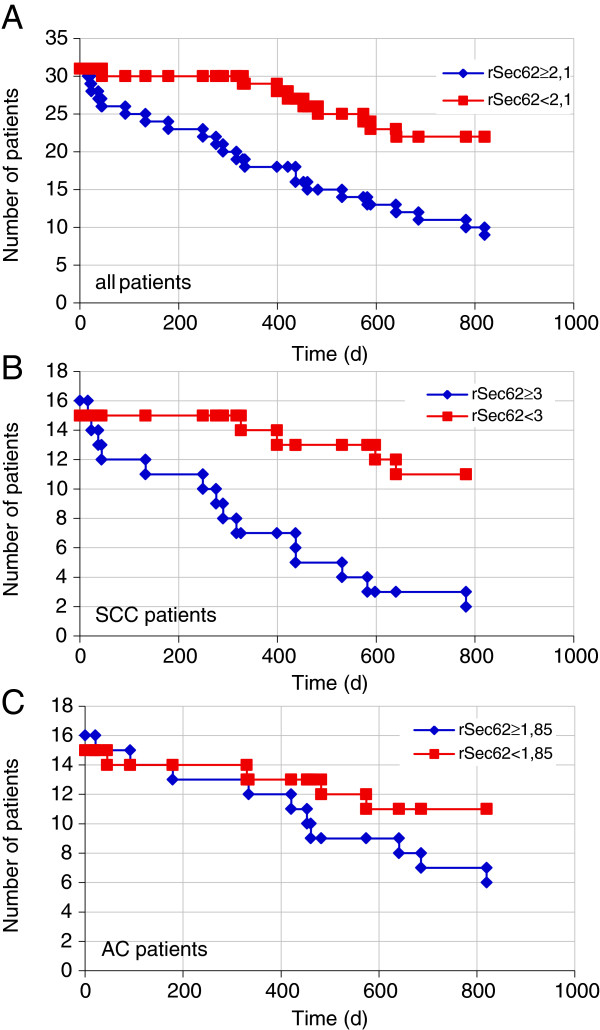
**Sec62 is a prognostic marker for NSCLC patients. A**, Patients with rSec62 < 2.1 exhibited significantly longer survival compared with those with rSec62 ≥ 2.1 (*P* < 0.001). **B**, The survival benefit of a low Sec62 protein level in the lung cancer tissue was significant in patients with SCC (*P* < 0.001). **C**, The survival benefit was not significant in patients with AC (*P* = 0.054).

In our previous study, we detected *SEC62* amplification and overexpression in NSCLC that did not correlate with patient age or sex but, at least for SCC, correlated with the appearance of lymph node metastases (higher Sec62 levels in N + tumors compared with N0 tumors) and the grade of differentiation (higher Sec62 levels in poorly differentiated G3 tumors compared with G2 tumors) [5]. Therefore, in the present study, we tested whether lower Sec62 levels in cancer tissue are associated with longer patient survival, which would indicate whether Sec62 can serve as a prognostic marker. We investigated the association between the rSec62 values of 70 NSCLC patients from our previous study [5] and these patients’ survival starting from the date of diagnosis. Patients were divided into two groups based on their rSec62 value using a threshold of 2.1 (all patients, Figure 1A), 3 (SCC patients, Figure 1B) and 1.85 (AC patients, Figure 1C), representing the median rSec62 value of the respective group. Survival analysis was visualized using Kaplan-Meier diagrams. Using the log-rank test, we observed a highly significant survival rate in the low rSec62 group compared with the high rSec62 group among all lung cancer patients, and SCC patients (*P* = 0.001 for all NSCLC patients, *P* = 0.001 for SCC patients, *P* = 0.054 for AC patients). The clinical relevance of the Sec62 protein level for SCC of the lung is even more important given that the increased Sec62 protein level also protects tumor cells from thapsigargin therapy [13].

### Treatment with calmodulin antagonists mimics changes in the cytosolic calcium concentration induced by SEC62 silencing

**Figure 2:**
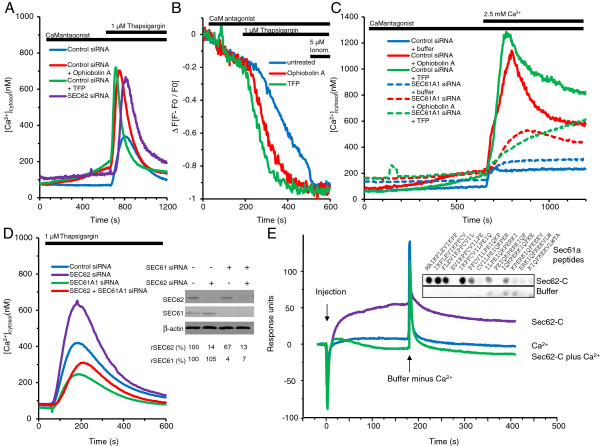
***SEC62 *silencing and calmodulin antagonists affect cellular calcium homeostasis similarly. A**, HeLa cells were transfected with *SEC62-* or control siRNA, after 96 h loaded with FURA2-AM, and subjected to Ca^2+^ imaging. After 60 s in Ca^2+^-free buffer, cells were treated with ophiobolin A (100 μM), TFP (10 μM), or buffer for 10 min, and then thapsigargin was added. The graphs represent the mean cytosolic Ca^2+^ concentration of 182 (control siRNA), 325 (control siRNA + ophiobolin A), 198 (control siRNA + TFP) or 82 cells (*SEC62* siRNA). **B**, HeLa-CES2 cells were loaded with Fluo5N-AM. After 60 s in Ca^2+^-free buffer, ophiobolin A, TFP, or buffer was added, samples were measured for 2 min, then thapsigargin and after 5 min ionomycin was added. **C**, HeLa cells were transfected with *SEC61A1-* or control siRNA, after 96h loaded with FURA2-AM, and after 60 s in Ca^2+^-free buffer treated with ophiobolin A, TFP or buffer. After 10 min Ca^2+^-solution was added (2.5 mM free Ca^2+^). **D**, HeLa cells were transfected with *SEC62*- , *SEC61A1*-, a combination of both siRNAs, or control siRNA, loaded with FURA2-AM, incubated in Ca^2+^-free buffer and subsequently treated with thapsigargin. The graphs represent the mean cytosolic Ca^2+^ concentration of 547 (control siRNA), 353 (*SEC62* siRNA), 495 (*SEC61A1* siRNA), or 395 cells (*SEC62* + *SEC61A1* siRNA). The insert shows the silencing efficiency determined by western blot (n=3). **E**, SPR spectroscopy was performed with Sec61α N-terminal peptide (measuring-cell) and TRAM N-terminal peptide (control-cell). Ca^2+^-containing buffer (control) or purified Sec62-C-His in buffer with or without Ca^2+^was passed over both cells. The insert shows a peptide spot binding assay. Peptides spanning the N-terminus of Sec61α were synthesized on a cellulose membrane and incubated with Sec62-C-His (1 μM) in binding buffer. Bound protein was detected using anti-His-POD coupled antibody and visualized with a luminescence-imaging system.

Previously, we have suggested *SEC62* silencing as a possibility for overcoming the protective effect of *SEC62* overexpression against thapsigargin, as *SEC62* silencing led to an increase in cytosolic Ca^2+^ and enhanced Ca^2+^ leakage from the ER in response to thapsigargin [13]. We also discovered a crucial influence of calmodulin on ER Ca^2+^ homeostasis; ER Ca^2+^ leakage is limited by Ca^2+^-dependent binding of calmodulin to the Sec61 complex [17,23]. The delivery of siRNAs for therapeutic applications is still problematic. Therefore, to determine whether Sec62 regulates calmodulin binding to the Sec61 complex or modulates the Sec61 complex, we examined the effects of the calmodulin antagonists, trifluoperazine (TFP) and ophiobolin A, on Ca^2+^ homeostasis compared with the effects of siRNA-mediated Sec62 depletion. Interestingly, all three approaches resulted in a comparable increase in cytosolic Ca^2+^ with or without thapsigargin treatment (Figure 2A). The results strongly suggest that a similar molecular mechanism leads to dysregulation of cellular Ca^2+^ homeostasis after *SEC62* silencing and after treatment with calmodulin antagonists.

To verify that indeed Ca^2+^ leakage from the ER is responsible for the increase in cytosolic Ca^2+^ concentration after treatment with ophiobolin A or TFP, we first used HeLa-CES2 cells in combination with Fluo5N to directly measure changes in ER luminal Ca^2+^. We observed an initial Ca^2+^-release from the ER after addition of calmodulin antagonists and a significantly higher efflux in the ophiobolin A or TFP pretreated cells in response to thapsigargin (Figure 2B), with τ_1/2_-values of 163 s for the buffer control, 87 s after pretreatment with ophiobolin A and 65 s after pretreatment with TFP. Next, we asked if the calmodulin antagonists influence the store operated calcium entry (SOCE). To this end, we measured the cytosolic Ca^2+^ concentration after treating the cells externally with a Ca^2+^-containing buffer instead of thapsigargin and EGTA. These experiments disclosed that SOCE was also significantly stimulated by pretreatment with calmodulin antagonists. Moreover, a comparison between cells treated with control siRNA and cells treated with two different siRNAs directed against *SEC61A1* indicated a crucial function of the Sec61 channel in SOCE under these conditions (Figure 2C). We note that we used a HeLa cell-based model system rather than lung cancer cells for two main reasons. First, the HeLa cells provide a well-established model system for *SEC61A1* or *SEC62* gene silencing, and live-cell Ca^2+^ imaging. Second, we were able to compare the results of live-cell Ca^2+^ imaging experiments on cells treated with *SEC61A1* or *SEC62* siRNA with our previous observations (Figure 2A–D) [13,24].

Furthermore, we examined whether the effect of Sec62 on ER Ca^2+^ leakage can be linked to the Ca^2+^-permeable Sec61 complex as has been previously shown for the effects of TFP and ophiobolin A [17,24]. To address this question, we treated HeLa cells for 96 h with *SEC62* siRNA, *SEC61A1* siRNA, *SEC62* plus *SEC61A1* siRNA, or a negative control siRNA. Simultaneous silencing of *SEC61A1* and *SEC62* by siRNA had an inhibitory effect on *SEC62* silencing-induced Ca^2+^ efflux (Figure 2D). Western blot analysis indicated that the silencing efficiency of both siRNAs was > 80% (Figure 2D, insert). Thus, calmodulin antagonists and Sec62 contribute to reducing Ca^2+^ leakage from the ER at the Sec61 complex level. As has already been shown for calmodulin [17], Sec62 presumably acts by direct interaction with Sec61α.

**Figure 3:**
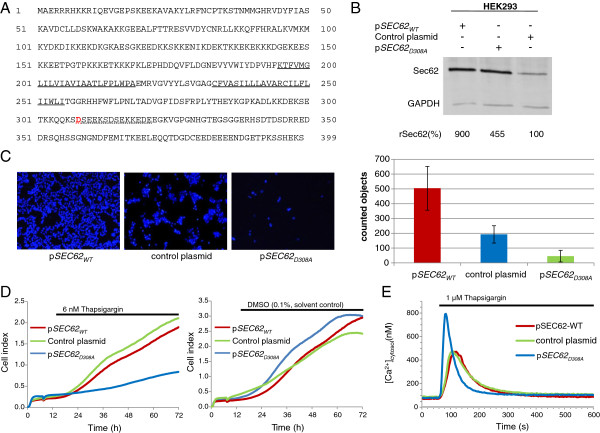
**A mutation in the putative EF hand motif of Sec62 affects cell migration, growth and ER calcium efflux in a dominant-negative manner. A**, Sequence of the human Sec62 protein. Transmembrane domains 1 and 2 are indicated with a solid underline. The predicted EF hand motif is indicated with a dotted underline. In the plasmid-encoded *SEC62*_*D308A*_, amino acid D308 (red) was replaced with an alanine. **B**, Sec62 protein levels in stably transfected HEK293 cells were analyzed by western blot analysis. **C**, HEK293 cells stably transfected with pIRES-GFP-*SEC62*-WT, pIRES-GFP-*SEC62*_*D308A*_ or pIRES-GFP (control plasmid) were seeded in normal growth medium without FBS in the top chamber of a BD-Falcon Fluoroblok migration system (24-well format). The lower chamber contained the same medium with 10% FBS as an attractant. After 72 h migrated cells were fixed with methanol and DAPI stained. Migration was analyzed by fluorescence microscopy using a 10-fold objective magnification. Migrated cells in at least five individual images were automatically counted using NIS-Elements AR Software (Nikon, Düsseldorf, Germany). The mean values and standard deviation are shown in the diagram. **D**, Stably transfected HEK293 cells (5 × 10^3^) were seeded in a 96-well ePlate and growth was examined in the xCELLigence RTCA system. After 300 min, 6 nM thapsigargin (left panel) or 0.1% DMSO (solvent control, right panel) was added to each well. All samples were measured in triplicate. **E**, Stably transfected HEK293 cells were seeded on glass slides in 6-cm dishes and loaded with FURA2-AM. Forty-five minutes later the cells were used for Ca^2+^ imaging as described in the legend for Figure 2. After 60 s incubation with EGTA buffer, the cells were treated with 1 μM thapsigargin. The curves shown in the diagram represent the mean cytosolic Ca^2+^ concentration of 158 cells (p*SEC62*-WT), 159 cells (p*SEC62*_*D308A*_) and 160 cells (control plasmid).

Peptide binding experiments were carried out to directly demonstrate the putative interaction of Sec62 with Sec61α and identify the Sec62 binding site. Peptide spots that correspond to the human Sec61α were synthesized on cellulose membranes. The peptides consisted of 12 amino acid residues and overlapped adjacent peptides by 10 residues. The peptides were incubated with the C-terminal cytosolic domain of the double-spanning membrane protein, Sec62. The C-terminal domain of Sec62 (Sec62C) preferentially bound to the N-terminal peptide of Sec61α (amino acid residues 1–16; Figure 2E, insert). In subsequent SPR spectroscopy analysis (Figure 2E), the interaction of Sec62C with the N-terminal peptide of Sec61α was confirmed. Sec62C showed more pronounced binding to Sec61α in the absence of Ca^2+^ than in its presence. *In silico* analysis of the Sec62 sequence (http://www.bioinformatics.org/calpred/ index.html) identified a potential EF hand in the C-terminal domain of vertebrate Sec62 (Figure 3A), which may explain this Ca^2+^ effect (amino acid residues 308–319, see below).

### Mutation in a predicted calcium-binding motif in the C-terminal domain of Sec62 leads to a dominant-negative effect on cell migration and ER calcium leakage

Previously, we showed that Sec62 depletion inhibits the spread of metastatic tumor cells and increases cell sensitivity to Ca^2+^-driven ER stress [12,13]. By introducing the D308A mutation into the predicted Ca^2+^-binding motif within the C-terminal domain of Sec62, we confirmed the function of Sec62 in regulating ER Ca^2+^ homeostasis (Figure 3A). In this experiment, the expression of plasmid-encoded *SEC62*-WT or *SEC62*_D308A_ was evaluated by quantitative western blot analysis of the stably transfected HEK293 cell lines. We observed a nine-fold increase in Sec62 in the presence of pSEC62-WT and an almost five-fold increase in Sec62 in the presence of p*SEC62*_*D308A*_ in comparison with the control plasmid (Figure 3B). We then compared stably transfected HEK293 cells overexpressing the plasmid-encoded mutant *SEC62* (p*SEC62*_*D308A*_-IRES-GFP) with cells overexpressing *SEC62-*WT (p*SEC62*-IRES-GFP). Overproduction of Sec62-WT led to increased migration, which is in agreement with our previous observations [5]. In contrast, overproduction of the mutant Sec62 protein, even in the presence of the endogenous Sec62-WT protein, reduced cell migration in a manner similar to *SEC62* silencing (Figure 3C). Also, the sensitivity to thapsigargin (Figure 3D) and thapsigargin-induced Ca^2+^ leakage from the ER increased after *SEC62*_D308A_ expression (Figure 3E). Overall, SEC62-WT overexpression did not affect cell growth or ER Ca^2+^ leakage, whereas *SEC62*_D308A_ overexpression led to a phenotype comparable to that of *SEC62* silencing. These experiments clearly indicate a direct influence of the predicted EF hand motif in Sec62 on ER Ca^2+^ homeostasis and its direct connection to the observed phenotypes.

### HeLa and HEK293 cells are more sensitive to TFP treatment than PC3 cells

**Figure 4:**
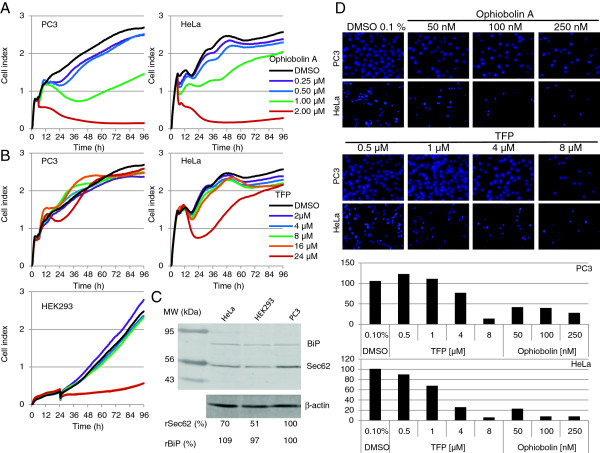
**Reduced *SEC62 *expression correlates with slightly increased sensitivity to TFP in PC3 cells. A**, Effect of ophiobolin A treatment on the growth of PC3 and HeLa cells. PC3 or HeLa cells (1 × 10^4^) were seeded in a 96-well ePlate and growth was examined by the xCELLigence RTCA system. After 330 min, cells were treated with buffer alone or buffer + ophiobolin A at the indicated concentrations. All samples were measured in triplicate. The cell index was normalized to the time point of cell treatment (330 min). **B**, The same analysis as described in A was performed on PC3, HeLa and HEK293 cells after treatment with TFP at the indicated concentrations. **C**, Quantification of the ER proteins, Sec62 and BiP, by western blot analysis. **D**, PC3 or HeLa cells were seeded in normal growth medium without FBS and supplemented with ophiobolin A, TFP or DMSO (control) at the indicated concentrations in the top chambers of a BD-Falcon Fluoroblok migration system (24-well format). The upper chambers were set in the lower chambers, which contained the same medium with 10% FBS as an attractant. After 72 h (PC3) or 24 h (HeLa), migrated cells were fixed with methanol and DAPI stained. Migration was analyzed by fluorescence microscopy. The quantitative data from this experiment are shown in the diagram.

To study the influence of TFP and ophiobolin A on cellular processes other than Ca^2+^ homeostasis, we analyzed the proliferation of PC3 and HeLa cells in the presence of these two calmodulin antagonists. We also analyzed HEK293 cells with respect to their TFP sensitivity. The main aim of this set of experiments was to determine the TFP and ophiobolin A concentrations that do not inhibit cell growth in subsequent cell migration or thapsigargin sensitivity studies. PC3 and HeLa cells exhibited the same sensitivity to ophiobolin A; both cell lines exhibited normal growth behavior up to a concentration of 500 nM, whereas higher concentrations significantly inhibited cell growth (Figure 4A). In contrast, PC3 cells tolerated TFP up to 24 μM, while HeLa cells exhibited a time-limited growth inhibition between 24 and 60 h after adding the compound, indicating that HeLa cells were more sensitive to TFP treatment than PC3 cells (Figure 4B). HEK293 cells exhibited normal proliferation with up to 8 μM of TFP in the medium, whereas cell growth was almost completely inhibited with higher concentrations. Based on these findings, we used concentrations of up to 250 nM of ophiobolin A and up to 8 μM of TFP as non-growth-inhibiting conditions for all cell lines in the subsequent experiments. Interestingly, the HeLa and HEK293 cells, which were more sensitive to TFP treatment, also expressed lower levels of Sec62 protein compared with PC3 cells. This difference was not because of a lower ER content, as the analyzed cell lines expressed similar levels of the ER chaperone, BiP (Figure 4C). The sensitivity of different cell lines to calmodulin antagonists may correlate with their specific Sec62 protein content, as indicated by our previous finding that Sec62 levels are crucial for cell tolerance against thapsigargin-induced ER stress [13]. The present findings affirmed the direct role of Sec62 in the cellular response to Ca^2+^-driven ER stress.

### Treatment with calmodulin antagonists and SEC62 silencing result in comparable cellular phenotypes

**Figure 5:**
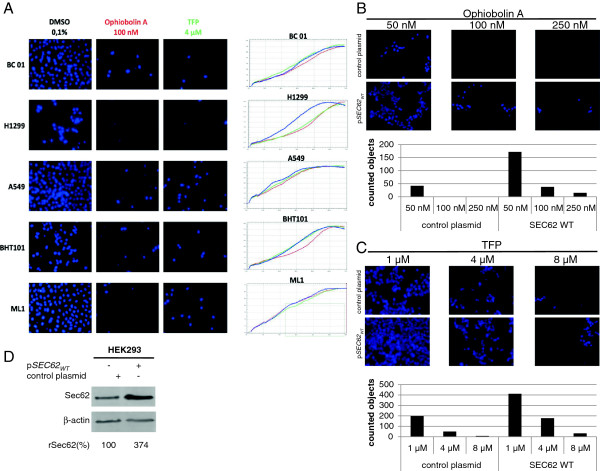
**Calmodulin antagonist treatment affects cell migration, which can be overcome by *SEC62 *overexpression. A**, Human lung (BC01, H1299 and A549) and thyroid cancer cells (BHT101 and ML1) were seeded in normal growth medium without FBS and treated with either ophiobolin A or TFP, and seeded in the top chamber of a BD-Falcon Fluoroblock migration system (24-well format). The upper chambers were set in the lower chambers, which contained the same medium with 10% FBS as an attractant. After 72 h, migrated cells were fixed with methanol and DAPI stained. Migration was analyzed by fluorescence microscopy. To exclude the possibility that the effects seen in the migration assay were caused by effects on cell proliferation, the cells were also analyzed in the xCELLigence system. To this end, 1 × 10^4^ cells were seeded in a 96-well ePlate and growth was examined using the RTCA software. Three hundred minutes after seeding, the cells were treated with either ophiobolin A (100 nM), TFP (4 μM) or DMSO (0.1%, solvent control). All samples were measured in triplicate. **B**, HEK293 cells stably transfected with a plasmid encoding wild-type *SEC62* (p*SEC62*-WT) or the respective control plasmid were seeded in normal growth medium without FBS and supplemented with TPA (10 nM) and ophiobolin A at the indicated concentrations in the top chamber. Migration was analyzed as described in A. **C**, The experiments described in B were performed in the presence of TFP instead of ophiobolin A at the indicated concentrations. **D**, Sec62 protein content was analyzed in stably transfected HEK293 cells by western blot analysis.

Next, we investigated whether a strongly reduced migration potential and increased sensitivity to thapsigargin-induced ER stress can also be caused by TFP or ophiobolin A treatment. First, the cell migration of PC3 and HeLa cells was examined in the presence of increasing amounts of ophiobolin A or TFP. We found a dose-dependent reduction in cell migration with both cell lines with both treatments (Figure 4D). Again, HeLa cells were more sensitive to the treatments than PC3 cells. To confirm the results, we tested different human lung (H1299, A549 and BC01) and thyroid cancer cell lines (BHT101 and ML1). We have previously reported reduced migration of these cell lines after transfection with *SEC62* siRNA [5]. Here, we found that 4 μM TFP and 100 nM ophiobolin A had the same effect on each cell line, strongly inhibiting cell migration without affecting cell proliferation (Figure 5A).

Because Sec62 depletion by siRNA transfection alone was sufficient to block cell migration in previous experiments [12], we tested whether *SEC62* overexpression can rescue ophiobolin A- or TFP-treated cells. We used HEK293 cells, which only poorly migrate without treatment but can be stimulated to migrate by the addition of 12-O-tetradecanoylphorbol 13-acetate (TPA), a drug that down-regulates agonist-driven Ca^2+^ release from the ER [25] and stimulates cell migration [26,27]. We compared HEK293 cells stably transfected with a pIRES-*GFP* vector (control plasmid) and HEK293 cells stably overexpressing plasmid-encoded *SEC62* (p*SEC62*-IRES-GPF). The migration of the control plasmid-transfected HEK293 cells was completely inhibited by 100 nM ophiobolin A or 8 μM TFP (Figure 5B and C). However, cells overexpressing *SEC62* still migrated under these conditions (Figure 5B and C), indicating that the Sec62 protein content resulted in higher cell resistance to treatment with calmodulin antagonists. Quantitative western blot analysis confirmed a four-fold increase in Sec62 in the p*SEC62*-WT-carrying HEK293 cells (Figure 5D). These observations support a Ca^2+^-dependent influence of Sec62 on cell migration.

### Growth inhibition induced by calmodulin antagonists is enhanced by Sec62 depletion

**Figure 6:**
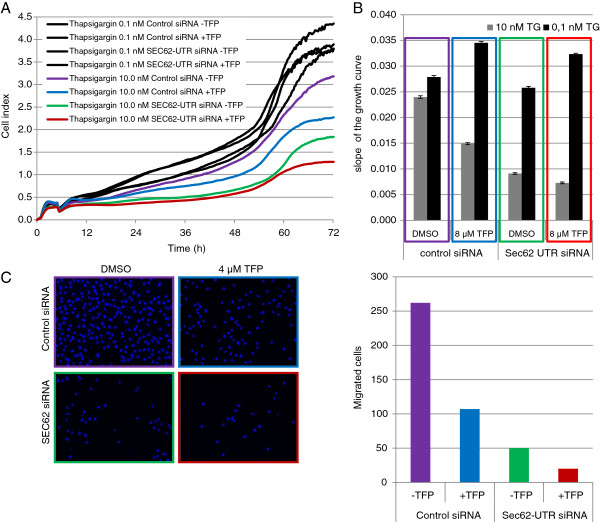
***SEC62 *silencing and TFP treatment additively affect cell growth and migration in PC3 cells. A**, Cells were seeded in 6-cm dishes and transfected with *SEC62* siRNA or control siRNA 24 h and 48 h after seeding. Twenty-four hours after the second transfection, 5 × 10^3^ PC3 cells were seeded in a 96-well ePlate and growth was examined using the xCELLigence RTCA system. After 300 min, the cells were treated with 10 nM thapsigargin or 0.1 nM thapsigargin in the presence of DMSO (0.1%, solvent control) or TFP (8 μM). All samples were measured in triplicate. **B**, The slopes of the growth curves shown in A between 8–72 h were calculated using the RTCA software. The error bars indicate standard deviations. **C**, Cells were treated with *SEC62* siRNA or control siRNA as described in A. Twenty-four hours after the second transfection, cells were seeded in normal growth medium without FBS and supplemented with either 4 μM TFP or 0.1% DMSO (control) in the top chamber of a BD-Falcon Fluoroblok migration system (24-well format). The lower chamber contained the same medium with 10% FBS as an attractant. After 72 h, migrated PC3 cells were fixed with methanol and DAPI stained. Migration was analyzed by fluorescence microscopy. **D**, Migrated cells from C were automatically counted using the NIS-Elements AR Software (Nikon).

Because treatment with calmodulin antagonists led to the same phenotype as Sec62 depletion with respect to cell migration, we next investigated whether this was also true for the increased thapsigargin sensitivity of the cells. PC3 cells were transfected with control siRNA or siRNA specifically directed against the *SEC62* mRNA, followed by treatment with 10 nM thapsigargin in the presence of 8 μM TFP or 0.1% DMSO (solvent control). Sec62-depleted cells exhibited greater sensitivity to thapsigargin and similar behavior to control siRNA-transfected cells after TFP treatment, indicating a slightly weaker decline in the growth rate (Figure 6A and B). Combined treatment with *SEC62* siRNA and 8 μM TFP resulted in even stronger growth inhibition, indicating an additive effect of *SEC62* silencing and calmodulin antagonist treatment (Figure 6A and B). This possible additive effect also appeared with respect to cell migration (Figure 6C and D). Taken together, these results indicate that growth inhibition by treatment with calmodulin antagonists and reduction in cellular Sec62 protein affect the same mechanisms, providing valuable hints regarding the function of Sec62 under cellular stress conditions.

## Discussion

### Sec62 as a new prognostic marker for NSCLC patients

Because *SEC62* silencing inhibits cancer cell migration and increases sensitivity to Ca^2+^-driven cellular stress, we investigated whether Sec62 represents not only a possible new target for anti-cancer therapies, but also a prognostic marker for lung cancer patients. A low rSec62 value predicts increased survival of NSCLC patients, with an even stronger predictive potential for SCC patients. Together with our previous findings that *SEC62* is overexpressed and correlates with lymph node metastasis (N + vs. N0) and cancer progression (G3 vs. G2) in SCC of the lung [5], the results indicate that Sec62 plays a crucial role in lung cancer biology and is both a promising new target for cancer therapy and a reliable marker of clinical outcomes. Additional studies are needed to determine whether the role of Sec62 as a prognostic marker is solely because of the tumor cells’ dependency on a sufficient Sec62 level to enable metastasis and resistance to Ca^2+^-driven cellular stress, or whether Sec62 has additional contributing functions.

### Phenotypic analogy of cellular calcium changes following treatment with calmodulin antagonists provides new insight into molecular events in Sec62-depleted cells

**Figure 7:**
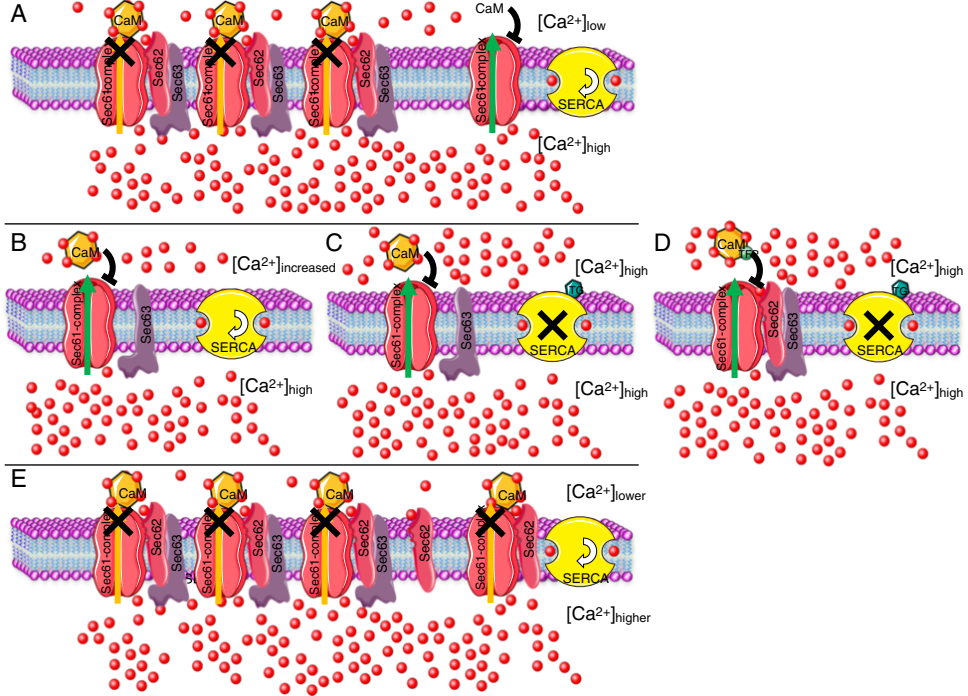
**Model for Sec62’s influence on ER Ca**^**2+ **^**efflux via the Sec61 complex under various conditions. A**, Physiological situation: most Sec61 complexes are associated with Sec62/Sec63-complexes. Calcium ions (Ca^2+^) leaking through the channel to the cytosol are detected by Sec62‘s EF hand, thus facilitating an interaction between Ca^2+^-CaM and the Sec61 complex and subsequent sealing of the channel. Sec62/Sec63-free Sec61 complexes allow a basal leakage of Ca^2+^, which is counteracted by SERCA activity. **B**, SEC62 knockdown conditions: depletion of Sec62 leads to a lack of calcium detection by Sec62 on the cytosolic surface of the ER. Ca^2+^-CaM is no longer recruited to the Sec61 complex and Ca^2+^ leakage persists, resulting in a slightly increased cytosolic Ca^2+^ concentration and a predisposition of the cells to apoptosis. Hence, Ca^2+^-dependent cell migration is inhibited. **C**, Compared with the situation described in B, the SERCA pump is inhibited by thapsigargin. The increased leakage combined with the inactivated back-pumping leads to a dramatic elevation in the cytosolic Ca^2+^ concentration and the cells undergo apoptosis. **D**, Trifluoperazine conditions: the situation described in B and C is mimicked by TFP treatment. Here, Sec62 still detects the leaking Ca^2+^, but the Ca^2+^-CaM-Sec61 complex interaction is blocked by TFP. In the absence of thapsigargin, the lack of interaction leads to the situation described in B; in the presence of thapsigargin, it leads to the situation described in C. **E**, Pathophysiological situation: an increased level of Sec62 protein probably leads to sealing of more Sec61 complexes, which may reduce the cytosolic Ca^2+^ level and/or increase the ER Ca^2+^ concentration, thereby protecting the cells against thapsigargin-induced ER stress.

We have previously reported strong inhibition of cell migration in different human cancer cells after Sec62 depletion by transfection with *SEC62* siRNA [5,12]. *SEC62* silencing markedly increased cell sensitivity to ER stress induced by dysregulation of cellular Ca^2+^ homeostasis, as shown by the more pronounced growth inhibition of Sec62-depleted cells after treatment with the SERCA inhibitor, thapsigargin, compared with control cells [5,13]. These results indicate that Sec62 plays a crucial role in cell migration and the ER stress response, particularly in cancer cells. However, we could not determine the molecular mechanisms responsible for these phenomena, as the function of Sec62 is only partially understood, even under physiological conditions, with some evidence for a role in protein transport at the ER of mammalian cells [9,10]. Sec62 could be involved in the transport of a particular subset of precursor proteins, including proteins that play crucial roles in cell migration and the ER stress response. However, we propose a model in which Sec62 influences these processes by regulating cellular Ca^2+^ homeostasis (Figure 7). This possibility is supported by the key role of Ca^2+^ in cell migration and ER stress [14,28-30], the potential EF hand motif in the cytosolic C-terminus of Sec62, the increase in basal cellular Ca^2+^ in response to *SEC62* silencing, and the markedly elevated cytosolic Ca^2+^ in response to thapsigargin treatment after *SEC62* silencing [13]. Though sparse evidence supports the first theory, Sec62’s influence on Ca^2+^ homeostasis is strongly supported by the present findings. Here, we showed that Sec62 depletion by siRNA transfection and treatment with calmodulin antagonists resulted in very similar changes in basal cellular Ca^2+^ levels and increased cytosolic Ca^2+^ concentrations after thapsigargin treatment. We also found that the treatment of different human cancer cells with calmodulin antagonists led to the same cellular phenotypes as observed after *SEC62* silencing, namely cell migration inhibition and markedly higher cell sensitivity to thapsigargin-induced ER stress. The crucial role of Sec62 in cellular Ca^2+^ homeostasis was further supported by the synergistic action of treatment with *SEC62* siRNA and calmodulin antagonists in regard to the sensitivity to thapsigargin-induced ER stress and by the rescue of cell migration by *SEC62* overexpression in cells pretreated with calmodulin antagonists.

Furthermore, the dominant-negative phenotype induced by mutation of the predicted EF hand motif in the Sec62 protein, which was completely congruent with the effects of Sec62 depletion or treatment with calmodulin antagonists, strongly points to a direct regulation of Sec62 function by Ca^2+^ binding to the motif. The Sec61 complex has recently been shown to form an important Ca^2+^ leakage channel in the ER, the major cellular Ca^2+^ reservoir, and that Ca^2+^ efflux via this polypeptide pore is regulated by calmodulin [23] and the ER luminal Hsp70 chaperone, BiP [31]. Taken together with our new findings that Ca^2+^ efflux from the ER after Sec62 depletion occurs through the Sec61 complex, we propose a model in which Sec62 is an additional regulator of the Sec61 Ca^2+^ leakage channel. Sec62 regulates Ca^2+^ leakage via a direct interaction with Sec61. The association of these two proteins has already been demonstrated [7,8] and has been found to be Ca^2+^ sensitive (Figure 2D). Following our model, Sec62 senses emanating Ca^2+^ via a microdomain in close proximity to the Sec61 channel. After Ca^2+^ binding, Sec62 binding to Sec61 is relieved, thereby uncovering the binding site and facilitating the binding of Ca^2+^-calmodulin to Sec61 on the cytosolic surface of the ER, leading to closure of the channel (Figure 7A and E). In this model, the Sec62 variant with the mutated EF hand (Sec62_D308A_) is no longer able to sense the emanating Ca^2+^, and thus closure of the Sec61 channel by Ca^2+^-calmodulin binding would not occur, which explains the increased Ca^2+^ response observed in our live-cell Ca^2+^ imaging experiments. An additional mode of action of Sec62 on the luminal side is possible via a role in the recruitment of BiP as a Ca^2+^ efflux-limiting factor via its interaction with the J-domain-containing Hsp40 protein, Sec63 [7,8,11,32].

### Mimicking the Sec62-depletion phenotype with small molecule treatment as a possible new therapeutic option for cancer patients

Previous studies have shown that Sec62 depletion by transfection with *SEC62* siRNA leads to cell migration inhibition and higher sensitivity to ER stress induced by Ca^2+^ dysregulation [5,12,13]. Therefore, *SEC62* silencing seems to provide a potential approach for cancer treatment, especially lung and thyroid cancer, as such treatment could lead to reduced metastatic spread of tumor cells and higher sensitivity to chemotherapies working via the induction of ER stress. However, despite intensive studies over the past few decades [33-36], RNA interference remains unfeasible for clinical treatment of human diseases, mainly because of toxic side effects and problems in achieving adequate concentrations in the target tissues [37]. Our present results provide a potential strategy for overcoming these problems with tumors that overproduce Sec62.

In the current study, we showed that treatment of different human cancer cells with calmodulin antagonists induced a Sec62-depletion phenotype, including cell migration inhibition and higher sensitivity to Ca^2+^-driven ER stress. The same effects on tumor cell biology can be expected by treating patients with these substances, which have already been intensively discussed as potential anti-metastatic and anti-proliferative drugs [38-43]. In particular, TFP appears to be a promising candidate for trials in animal models, and in human patients, because it has previously been used as an antipsychotic and antiemetic drug [44,45]. Treatment with calmodulin antagonists could also provide the means for overcoming problems with treating patients with high levels of Sec62 protein in tumor cells [13]; here, a personalized therapeutic approach that also targets the SERCA pump using thapsigargin or tissue-specific peptide conjugates of thapsigargin appears to be promising [46-50]. Based on the present results, we propose combined treatment with TFP and targeted thapsigargin as a powerful new strategy for treating patients with SCC of the lung (Figure 7D), which is especially important because the therapeutic options for this malignancy are very limited and increased levels of Sec62 are a significant disadvantage in regard to survival.

## Conclusions

The present study describes a new function of Sec62 in regulating the calmodulin-mediated sealing of the Sec61 Ca^2+^ leakage channel in the ER, which may explain how the up-regulation of *SEC62* expression results in reduced survival among lung cancer patients. Furthermore, it provides the first molecular insight into the mechanism of resistance of Sec62-overproducing tumor cells to treatment with thapsigargin. Using calmodulin antagonists, including TFP, we can inhibit cancer cell migration and overcome the problem of Sec62 overproduction in response to thapsigargin, which may also improve the treatment of these cancer entities in future combinatorial therapeutic strategies.

## Competing interests

The authors’ declare no potential conflicts of interest with respect to the research, authorship, and/or publication of this article.

## Authors’ contributions

ML performed Ca^2+^ imaging, cell migration and real-time cell analysis experiments using the Sec62_*D308A*_ variant (Figure 3), the human thyroid and lung cancer cell lines (Figure 5A), compared Sec62 levels in different cell lines by western blot analysis (Figure 4C), and participated in writing the manuscript. SS generated the point mutation in *SEC62* and performed Ca^2+^ imaging experiments with combined knockdown of *SEC61A1* and *SEC62* (Figure 2D). NS performed Ca^2+^ imaging experiments with calmodulin antagonists (Figure 2A), measurements of ER lumenal Ca^2+^ (Figure 2B) and of SOCE (Figure 2C). MJ performed protein-peptide interaction studies (Figure 2D). JL, FL and HJS analyzed the clinical data and performed statistical analysis (Figure 1). AC supervised all Ca^2+^ imaging experiments. RZ supervised all cell biological experiments and participated in writing the manuscript. MG performed real-time cell analysis (Figure 4 and 6), cell migration analysis (Figure 5 and 6), generated the stable HEK293 p*SEC62*-IRES-GFP and pIRES-GFP cell lines and participated in writing the manuscript. All authors read and approved the final manuscript.

## Pre-publication history

The pre-publication history for this paper can be accessed here:

http://www.biomedcentral.com/1471-2407/13/574/prepub
